# Unhealthy family functioning is strongly associated with warfighter brain health following traumatic brain injury in United States service members and veterans

**DOI:** 10.3389/fneur.2025.1475098

**Published:** 2025-08-12

**Authors:** Tracey A. Brickell, Louis M. French, Brian J. Ivins, Sara M. Lippa, Megan M. Wright, Samantha M. Baschenis, Jamie K. Sullivan, Lars D. Hungerford, Rael T. Lange

**Affiliations:** ^1^Traumatic Brain Injury Center of Excellence, Walter Reed National Military Medical Center, Bethesda, MD, United States; ^2^National Intrepid Center of Excellence, Walter Reed National Military Medical Center, Bethesda, MD, United States; ^3^Uniformed Services University of the Health Sciences, Bethesda, MD, United States; ^4^Contractor, General Dynamics Information Technology, Silver Spring, MD, United States; ^5^Traumatic Brain Injury Center of Excellence, Silver Spring, MD, United States; ^6^Contractor, CICONIX, Annapolis, MD, United States; ^7^Traumatic Brain Injury Center of Excellence, Naval Medical Center San Diego, CA, United States; ^8^Traumatic Brain Injury Center of Excellence, Fort Carson, Colorado Springs, CO, United States

**Keywords:** family functioning dysfunction conflict, military service member veteran warfighter, traumatic brain injury, brain health, neurobehavioral, posttraumatic stress

## Abstract

**Objective:**

To examine the relationship between family functioning and health-related quality of life (HRQOL) outcomes following traumatic brain injury (TBI) in service members and veterans (SMVs).

**Participants:**

Participants were 359 United States SMVs classified into three groups: non-injured controls (NIC, *n* = 62); uncomplicated mild TBI (MTBI; *n* = 189); and complicated mild, moderate, severe, and penetrating TBI (STBI; *n* = 108). Participants completed 10 HRQOL measures from the TBI-QOL and Neuro-QOL, and the Family Assessment Device-General Functioning subscale (FAD-GF) 2-or-more years post-injury. Using the FAD-GF, the NIC, MTBI, and STBI participants were divided into six subgroups: Group 1 = NIC Healthy Family Functioning (HFF) (*n* = 34); Group 2 = NIC Unhealthy Family Functioning (UnHFF) (*n* = 28); Group 3 = MTBI HFF (*n* = 88); Group 4 = MTBI UnHFF (*n* = 101); Group 5 = STBI HFF (*n* = 58); and Group 6 = STBI UnHFF (*n* = 50).

**Results:**

Participants with UnHFF had a significant and meaningfully higher number of clinically elevated HRQOL scores compared to those with HFF in the MTBI (*p* < 0.001, $\eta_{p}^{2}$ =0.07) and STBI (*p* = 0.001, $\eta_{p}^{2}$ =0.10) groups, but not in the NIC group (*p* = 0.107, $\eta_{p}^{2}$ =0.04). There were no differences in the total number of clinically elevated HRQOL scores when comparing the MTBI and STBI HFF groups to controls (*p* = 0.074 to 0.841). The MTBI and STBI UnHFF groups had a significant and meaningfully higher number of clinically elevated HRQOL scores when compared to controls (*p* < 0.001 to *p* = 0.018; $\eta_{p}^{2}$ =0.07 to.14). The MTBI UnHFF group was 10 to 28 times more likely to have poor HRQOL outcome compared to controls. The STBI UnHFF group was 6 to 17 times more likely to have poor HRQOL outcome compared to controls.

**Conclusion:**

UnHFF was strongly associated with poor long-term HRQOL. HFF was strongly associated with good long-term HRQOL outcome. Assessment and management of family distress may facilitate better TBI recovery and readiness in warfighters.

## Introduction

Military families face unique challenges such as frequent and lengthy periods of separation during deployment, and renegotiation of roles and relationships during reintegration. Reintegration can be more challenging when service members return with physical and mental health problems (1). Traumatic brain injury (TBI) is a common combat and noncombat injury in United States military personnel with over 80% classified as mild severity (2). Long-term complications are more likely following a moderate, severe, or penetrating TBI. A complicated mild TBI (MTBI) is often differentiated from an uncomplicated MTBI based on the presence of day-of-injury intracranial abnormality. Recovery following a complicated MTBI is thought to be to be similar to a moderate TBI, although the findings have been mixed (3–6). Neurobehavioral symptoms related to an uncomplicated MTBI should typically resolve within a few weeks and not newly develop, get worse, or be cyclical in the months or years post-injury. However, some service members and veterans (SMVs) report symptoms long after an uncomplicated MTBI, and fail to reach desirable fitness for duty and health-related quality of life (HRQOL) outcomes (7, 8). Physical and mental health conditions frequently co-occur, and may be associated with the TBI event itself or present pre-or post-injury (9, 10). Many SMVs have reported neurobehavioral and HRQOL symptoms up to 10 years following a TBI of any severity, which were largely accounted for by mental health symptoms, particularly posttraumatic stress (8, 11, 12).

TBI and mental health conditions are often referred to as *invisible injuries*, because their symptoms are more difficult to associate with a medical condition compared to injuries with conditions that are readily observable (e.g., bandages, limb loss, scars, prosthetic, wheelchair). Invisible injuries can complicate the family’s understanding of why the SMV is experiencing difficulty resuming daily activities and reestablishing emotional bonds (13). TBI and mental health conditions have been associated with higher levels of conflict and dysfunction in military families. In behavioral health treatment seeking SMVs, a majority reported some type of family problem (e.g., feeling like a guest in their home, children being afraid of them, disagreements over roles and responsibilities, marital discord, shouting/pushing/shoving) (14). SMVs with a diagnosis of depression or posttraumatic stress disorder (PTSD) were five times more likely to have family problems compared to SMVs without depression or PTSD. Emotional withdrawal, avoidance, and anxious symptoms were strongly associated with family problems. In treatment seeking SMVs at an outpatient clinic for SMVs with PTSD, TBI, and other mental health conditions, scores for family functioning were on average in the unhealthy range. Unhealthy family functioning has been related to lower parenting confidence and PTSD symptoms related to emotional numbing and avoidance (15, 16). Parents of military connected children (SMVs and co-parents) with higher levels of stress, depression, and anxiety and lower parenting confidence reported worse family functioning (17). Worse family functioning was reported by SMVs with a TBI compared to SMVs without TBI. Having an unclassified TBI severity in the medical records, a comorbid condition, combat experiences, and several sociodemographic characteristics (e.g., age, race, income) were related to worse family functioning, but not a moderate/severe TBI severity versus MTBI severity (18). Elsewhere, SMV neurobehavioral symptoms were associated with family functioning as reported by the SMV’s intimate partner, but not sociodemographic (age, sex, household income), TBI (TBI severity, years’ post-injury) comorbid diagnosis (PTSD, depression, pain, headaches), or military characteristics (military status, combat deployments, and combat exposure) (19).

Within the research literature there has been little consideration to the influence of family distress as a risk factor for chronic symptoms in SMVs with TBI. Worse neurobehavioral symptoms were reported by intimate partners of SMVs with TBI who reported higher levels of unhealthy family functioning and dissatisfaction in their couples relationship (19, 20). It is possible that when a service member recovers in an environment with high family conflict and dysfunction, their recovery will be compromised because conflict, disorganization, and poor affective and behavioral regulation and accommodations within their family may impede the care and support needed to recover and return to duty. Poor recovery following a TBI increases the probability that a warfighter may not return to the operational environment and may require medical separation, or may return with reduced cognitive-behavioral capabilities and degrade unit readiness (21).

The purpose of the current study was to examine the relationship between SMV-reported family functioning and HRQOL outcomes following a TBI of all severities and in non-injured controls. This study had three hypotheses. First, unhealthy family functioning would be associated with worse HRQOL outcomes following TBI and in the non-injured control group. Second, healthy family functioning would be associated with good long-term HRQOL outcome from TBI; i.e., TBI participants with healthy family functioning would report HRQOL outcomes that are similar to non-injured controls. Third, unhealthy family functioning would be associated with poor long-term HRQOL outcome from TBI; i.e., TBI participants with unhealthy family functioning would report worse HRQOL outcomes compared to non-injured controls.

## Materials and methods

### Participants

Participants were 359 United States SMVs prospectively enrolled in the 15-year longitudinal Natural History of TBI Study (NH Study).^1^ SMVs were recruited from outpatient clinics and inpatient wards from the Walter Reed National Military Medical Center (WRNMMC, 68.4%) and the Naval Medical Center San Diego (9.7%). Service members were approached in an appropriate location (e.g., waiting room). The remainder were recruited via community-based recruitment outreach (21.9%), such as a study investigator attending events held across the United States, nationally-based military organizations posting on social media, and displaying study flyers or business cards at military treatment facilities. Interested participants could volunteer to leave their contact details with a study investigator or initiate contact themselves via a study email/phone number. A study investigator would then follow up with the participant and obtain written consent.

Participants were classified into three groups: non-injured controls (NIC, n = 62); uncomplicated mild TBI (MTBI; n = 189); and complicated mild, moderate, severe, and penetrating TBI combined (STBI; n = 108). Participants were included in the MTBI group if they met the following criteria for uncomplicated mild TBI: (i) Glasgow Coma Scale (GCS) = 13–15, post-traumatic amnesia (PTA) < 24 h, Loss of Consciousness (LOC) < 30 min, and/or alteration of consciousness (AOC) present, and (ii) no trauma-related intracranial abnormality on CT or MRI. Participants were included in the STBI group if they met one of the following criteria for [a] complicated mild TBI: (i) GCS = 13–15, PTA < 24 h, LOC < 30 min, and/or AOC present, and (ii) trauma-related intracranial abnormality on CT or MRI; [b] moderate TBI: LOC 1–24 h, PTA 1–7 days, and ICA present or absent; [c] severe TBI: LOC > 24 h, PTA > 7 days, and ICA present or absent, or [d] penetrating TBI: a breach of the cranial vault and/or dura mater by an external object (e.g., bullet, shrapnel) and/or skull fragment (i.e., skull fracture). Participants were included in the NIC group if they had no history of an orthopedic and/or soft-tissue injury, and no history of TBI. General exclusion criteria included being less than 18 years of age, lack of proficiency in conversational English, and a history of significant neurological or psychiatric conditions unrelated to the injury event or deployment (e.g., Meningioma, Bipolar Disorder).

SMVs were selected from a larger sample of participants that had been enrolled in the NH Study between February 2022 and December 2023 if they (a) had scored below the recommended cutoff on a measure designed to evaluate symptom over-reporting [see Measures section], (b) they had been evaluated 2-or-more years’ post-injury [TBI group only], (c) had completed all measures with no missing items, and (d) they could be confidently classified into either the NIC, MTBI or STBI groups.

The protocol under which these data were collected was approved by the WRNMMC Institutional Review Board. This study was completed in accordance with the guidelines of the Declaration of Helsinki. The study was undertaken with the understanding and written consent of each participant.

### TBI diagnosis and classification

Diagnosis and classification of TBI severity was based on a medical record review and comprehensive lifetime TBI history interview. The lifetime TBI history interview was completed by Masters-level clinical research personnel who were specifically trained by subject matter experts to evaluate the presence and severity of TBI. The TBI history interview consisted of the Ohio State University TBI identification method (22) and an extended semi-structured clinical interview designed to (a) extract more detailed information to estimate the presence/duration of LOC, PTA, AOC, and retrograde amnesia, and (b) gather military-specific information regarding injury circumstances (e.g., type of blast, protection worn). Final determination and classification of TBI severity was undertaken by consensus, giving consideration to all information, during case conferencing with subject matter experts trained in TBI diagnostic interviewing.

### Measures

#### Family functioning

The Family Assessment Device-General Functioning (FAD-GF) (23) is a 12-item short form of the larger Family Assessment Device that is designed to assesses the structural, organizational, and transactional characteristics of families across six dimensions of family functioning including problem solving, communication, roles, affective responsiveness, affective involvement, and behavior control. The FAD-GF has been widely used in both research and clinical practice to screen and identify families experiencing problems, and assess change following treatment, including military families (16, 17, 19, 24, 25). The measure is part of the National Institute of Neurological Disorders and Stroke (NINDS) Common Data Elements (CDE) in TBI research (26), and recommended for use in research examining the relationship of family interactions and recovery trajectory following TBI (27). Scores across all response items were averaged with a possible range from 1.00 (healthy) to 4.00 (unhealthy). A cut-score greater than 2.00 was established by the developers to be used for categorical analyses and to satisfy both the theoretical and content perspectives of the measure. The cut-score was revalidated more recently in a community sample and family therapy treatment seeking sample (28). A score ≥2 was classified as Unhealthy Family Functioning (e.g., *there are lots of bad feelings in our family*) and a score <2 was classified as Healthy Family Functioning (e.g., *in times of crisis we can turn to each other for support*) (29). The FAD-GF was used to classify the MTBI, STBI, and NIC participants into six family functioning groups as described below.

#### Health-related quality of life

The Traumatic Brain Injury Quality of Life (TBI-QOL) measurement system was developed and validated specifically for individuals with TBI using advanced psychometrics and development technology following the Patient-Reported Outcomes Measurement Information System (PROMIS) development standards (30). The measure is part of the NINDS CDEs in TBI research (26). Short form measures from psychological (Anxiety, Depression, Anger, Emotional and Behavioral Dyscontrol), physical (Fatigue, Headaches, Pain Interference), Cognitive (Cognition-General Concerns), and social (Ability to Participate in Social Roles and Activities) HRQOL domains were completed. In addition, the Sleep Disturbance scale from the Quality of Life in Neurological Disorders scale (Neuro-QOL) was completed (31). The Neuro-QOL was developed for individuals with neurological disorders neurological conditions or disorders such as stroke, multiple sclerosis, amyotrophic lateral sclerosis, Parkinson’s disease, epilepsy, and muscular dystrophy. For all HRQOL scales, a total raw score for each scale was calculated and converted to a T-score (*M* = 50, SD = 10). Higher scores reflected worse functioning except for Cognition-General Concerns and Ability to Participate in Social Roles and Activities. In addition, scores on each HRQOL measure were classified as clinically elevated using a cut-score of greater than one standard deviation from the mean (e.g., Anxiety >60 T; Cognition-General Concerns <40 T). The total number of clinically elevated scores across the 10 HRQOL measures was calculated (max = 10). Note that the TBI-QOL uses a TBI population as the normative group. As such, interpretation of T-scores is relative to a sample of individuals with TBI which may have some limitations for use in a non-TBI sample. Nonetheless, the TBI-QOL is recommended for research purposes (32) where the inclusion of non-TBI controls is considered mandatory. Use of the TBI-QOL with TBI and non-TBI control samples has been used in previous research (11, 33, 34). In addition, the clinical utility and psychometric properties of the TBI-QOL have been established in a TBI and non-TBI population (35).

#### SMV symptom validity

The Validity-10 (36) is a symptom validity test (SVT) designed to detect symptom exaggeration when administering the Neurobehavioral Symptom Inventory (NSI) (37). Clinical validation studies have supported its use for this purpose (38, 39). The Validity-10 scale consists of 10 items from the NSI that are considered atypical and infrequently endorsed by individuals following TBI. A cutoff score of >22 was used to classify symptom exaggeration (i.e., SVT Fail) (36).

#### Covariate

Concurrent PTSD and TBI are very common in SMVs. Previous literature has demonstrated the strong association of PTSD with poor long-term neurobehavioral and HRQOL outcome following a TBI of all severities. PTSD has been found to be one of the most significant and influential factors related to TBI outcomes, even more than TBI severity itself (11). As such, PTSD was used as a covariate in the current study. The 17-item PTSD Checklist-Civilian version (PCLC) (40) evaluated self-reported PTSD symptoms per the DSM-IV-TR (41) and was used as a covariate in statistical analyses. A total score can be calculated by summing all of the items (range = 17–85). For the purposes of this study, an alternate total score (i.e., PCLC Subtotal) was calculated by summing items that are considered unique to PTSD (e.g., repeated disturbing dreams of a stressful event). PCLC items selected included symptoms related to the Intrusions (1–5), Avoidance (6, 7), and Hyperarousal (16, 17) clusters identified in a meta-analysis (42), and supported by the National Center for PTSD, U. S Department of Veterans Affairs (www.ptsd.va.gov/understand/related/tbi_ptsd.asp). Higher scores reflected more severe PTSD symptoms.

### Family functioning group classification

For the purpose of this study, participants were categorized into six family functioning groups based on [a] their injury group (i.e., NIC, MTBI, STBI), and [b] Healthy Family Functioning (HFF) versus Unhealthy Family Functioning (UnHFF) classification on the FAD-GF. The resulting six groups were as follows: Group 1 = NIC HFF (*n* = 34); Group 2 = NIC UnHFF (*n* = 28); Group 3 = MTBI HFF (*n* = 88); Group 4 = MTBI UnHFF (*n* = 101); Group 5 = STBI HFF (*n* = 58); and Group 6 = STBI UnHFF (*n* = 50).

The breakdown of TBI severity in the STBI group, stratified by FAD-GF category, was as follows: (a) STBI HFF: complicated mild TBI (*n* = 18, 31.0%), moderate TBI (n = 15, 25.8%), severe TBI (*n* = 10, 17.2%), and penetrating TBI (*n* = 15, 25.9%); and (b) STBI UnHFF: complicated mild TBI (*n* = 11, 22.0%), moderate TBI (*n* = 11, 22.0%), severe TBI (*n* = 12, 24.0%), and penetrating TBI (*n* = 15, 32.0%).

### Statistical analysis

Statistical analysis was undertaken using SPSS 27.0. Descriptive statistics and group comparisons across the six family functioning groups for select demographic and injury variables (i.e., age, education, gender, ethnicity, time since injury, and PTSD symptoms [i.e., PCLC Subtotal)] was undertaken using ANOVA with posthoc analyses (continuous variables) or Chi-square analysis (categorical variables) where appropriate. Pearson correlation analysis (continuous variables) or ANOVA (categorical variables) was then used to examine the relationship between demographic and injury variables with the 10 HRQOL outcome measures in the entire group. Any demographic or injury variable that was meaningfully associated with the HRQOL measures (i.e., r_s_ ≥ 0.30 for continuous variables, or *p* < 0.05 for categorical variables) (43) was used as a covariate in subsequent analyses.

Descriptive statistics and group comparisons across the six family functioning groups for the 10 HRQOL scores, and the total number of clinically elevated HRQOL scores, was undertaken using ANCOVA (using PCL-C total 1–8 as a covariate). Pairwise comparisons were further undertaken using ANCOVA and partial eta squared effect sizes ( $\eta_{p}^{2}$ ). In order to reduce the number of pairwise comparisons, only select pairwise comparisons were undertaken to examine the three hypotheses as follows:

First, in order to examine the influence of family functioning on HRQOL outcome (Hypothesis 1), participants with healthy versus unhealthy family functioning (HFF vs. UnHFF) were compared in the NIC, MTBI, and STBI groups separately (i.e., NIC HFF vs. UnHFF [Group 1 v 2]; MTBI HFF vs. UnHFF [Group 3 v 4]; STBI HFF vs. UnHFF [Group 5 v 6]).
Second, in order to examine the influence of healthy family functioning on HRQOL outcome from TBI (Hypothesis 2), the MTBI HFF and STBI HFF groups were compared to the two NIC HFF and UnHFF groups (i.e., MTBI HFF vs. NIC UnHFF [Group 2 v 3]; STBI HFF vs. NIC UnHFF [Group 2 v 5]; MTBI HFF vs. NIC HFF [Group 1 v 3]; STBI HFF vs. NIC HFF [Group 1 v 5]).
Third, in order to examine the influence of unhealthy family functioning on HRQOL outcome from TBI (Hypothesis 3), the MTBI UnHFF and STBI UnHFF groups were compared to the two NIC HFF and UnHFF groups (i.e., MTBI UnHFF vs. NIC HFF [Group 1 v 4]; STBI UnHFF vs. NIC HFF [Group 1 v 6]; MTBI UnHFF vs. NIC UnHFF [Group 2 v 4]; STBI UnHFF vs. NIC UnHFF [Group 2 v 6]). In addition, odds ratios were calculated to further examine the influence of UnHFF on poor HRQOL outcome. Poor outcome was defined as 3-or-more clinically elevated HRQOL scores.

It is acknowledged that the probability of Type 1 error increases when multiple statistical comparisons are made. An adjusted *p*-value or a stepdown statistical procedure is often employed to control the false discovery rate for each comparison. However, a small sample size can undermine significance with medium-large effect sizes after controlling the false discovery and adjusting the significance criterion. Statistical significance can be a problematic measure of group differences because it is driven by many factors that can offset each other, such as effect size, sample size, and measurement variability. Since clinical research is often confounded by these factors, statistical significance is often of limited value. To improve the interpretation of significance, researchers commonly reply on clinically-relevant information such as effect sizes over adjustment to *p*-values. Effect sizes present the magnitude of the effect difference in outcomes between groups (44–46). In the current study, pairwise comparisons were interpreted as meaningful when both *p* < 0.05 and an effect size was medium or higher (i.e., $\eta_{p}^{2}$ ≥0.06).

## Results

Descriptive statistics for demographic and injury variables across the six family functioning groups is presented in Table 1. There were significant main effects for age (*p* = 0.026), education (*p* = 0.004), ethnicity (*p* = 0.028), and PCLC Subtotal (*p* < 0.001); but not for time since injury (*p* = 0.407) or gender (*p* = 0.883). Posthoc analyses revealed that Group 1 (NIC HFF) was older in age compared to Group 5 (STBI HFF; *p* = 0.014); and Group 1 had a higher number of years of education compared to Group 4 (MTBI UnHFF; *p* = 0.038), Group 5 (*p* = 0.008), and Group 6 (STBI UnHFF; *p* = 0.018). In addition, Group 1 had a lower proportion of participants who identified as being of white ethnicity compared to Group 3 (MTBI HFF; *p* = 0.017) and Group 5 (*p* = 0.003). For PCLC Subtotal, Group 4 had more severe PTSD symptoms compared to Groups 1, 3, and 5 (all *p*’s < 0.001), Group 2 (*p* = 0.048), and Group 6 (*p* = 0.020).

**Table 1:** Descriptive statistics for select demographic and injury variables by family functioning group.

Demographic and Injury variables	Group 1 NIC HFF		Group 2 NIC UnHFF		Group 3 MTBI HFF		Group 4 MTBI UnHFF		Group 5 STBI HFF		Group 6 STBI UnHFF	
	*M*	SD	*M*	SD	*M*	SD	*M*	SD	*M*	SD	*M*	SD
Age (years)^1^	47.7_a_	9.3	45.8	10.9	43.3	8.8	43.5	9.0	40.9_a_	8.8	43.9	10.8
Education (years)	16.7_abc_	2.2	16.1	2.5	15.8	2.2	15.3_a_	2.3	15.0_b_	2.3	15.1_c_	2.1
Time since injury (months)	–	–	–	–	14.2	5.8	13.9	5.6	12.7	4.7	13.5	5.3
PCLC Subtotal (raw score)	13.1_a_	6.0	15.9_b_	6.7	15.3_c_	6.5	20.2_abcde_	7.9	13.6_d_	5.9	16.4_e_	6.5
	%		%		%		%		%		%	
Gender: Male	94.1		92.9		94.3		95.0		98.3		94.0	
Ethnicity: White	64.5_ab_		72.0		85.9_a_		78.8		92.2_b_		78.3	

*N* = 359 (Group 1 = 34, Group 2 = 28, Group 3 = 88, Group 4 = 101, Group 5 = 58, Group 6 = 50). ^abcd^Same subscript denotes significant differences. PCLC, post-traumatic stress disorder checklist; NIC, non-injured controls; MTBI, uncomplicated mild TBI; STBI, combines severe TBI, moderate TBI, and complicated mild TBI. HFF, Healthy Family Functioning; UnHFF, Unhealthy Family Functioning; PCLC Subtotal, sum of PCLC items 1–7, 16, and 17.

Examination of the relationship between select demographic/injury variables and the 10 HRQOL outcome measures revealed no meaningful associations for age (range: r = 0.01 to r = 0.06), education (range: r = 0.04 to r = 0.18) or ethnicity (*p* = 0.129–0.987; except Ability to Participate in Social Roles and Activities [*p* = 0.049, d = 0.28]). However, there was a significant and strong relationship between PCLC Subtotal and all 10 HRQOL measures (all *p*’s < 0.001; r = 0.46 to r = 0.69). As such, PCLC Subtotal was used as a covariate in all subsequent analyses.

**Figure 1:**
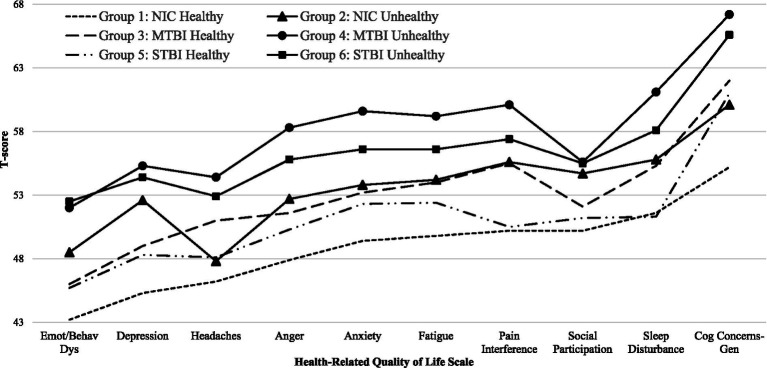
T-scores for health-related quality of life measures by family functioning group.

Descriptive statistics for the 10 HRQOL measures, and the total number of clinically elevated HRQOL scores, by group is presented in Table 2 and Figure 1. After controlling for PTSD, there were significant main effects across the six groups for the total number of clinically elevated HRQOL scores (*p* < 0.001), and for all 10 HRQOL measures [all p’s < 0.001 except Anxiety (*p* = 0.011)].

**Table 2:** Descriptive statistics for the health-related quality of life measures by family functioning group

Health-related quality of life measures	Group 1 NIC HFF		Group 2 NIC UnHFF		Group 3 MTBI HFF		Group 4 MTBI UnHFF		Group 5 STBI HFF		Group 6 STBI UnHFF	
	*M*	SD	*M*	SD	*M*	SD	*M*	SD	*M*	SD	*M*	SD
Anxiety^1^	49.4	9.3	53.8	8.5	53.2	9.3	59.6	7.2	52.3	9.2	56.6	7.4
Depression	45.3	7.1	52.6	6.5	49.0	7.8	55.3	7.1	48.3	8.1	54.4	7.5
Anger	47.9	7.6	52.7	9.0	51.6	9.1	58.3	9.0	50.3	8.0	55.8	8.5
Emot/Behav Dys	43.2	7.0	48.5	7.3	46.0	7.8	52.0	8.3	45.7	7.6	52.5	7.7
Fatigue	49.8	8.1	54.2	8.1	54.0	8.0	59.2	7.0	52.4	7.5	56.6	6.7
Sleep disturbance	51.6	9.6	55.8	9.2	55.3	9.3	61.1	7.6	51.3	9.0	58.1	7.9
Headaches	46.2	7.6	47.8	7.2	51.0	7.4	54.4	6.4	48.1	7.4	52.9	6.4
Pain interference	50.2	9.9	55.6	6.2	55.5	8.5	60.1	6.4	50.5	8.8	57.4	8.0
Cognition-general concerns	44.8	8.8	39.9	7.5	38.0	9.0	32.8	7.0	39.0	8.8	34.4	6.4
Social participation^a^	49.8	4.9	45.3	5.8	47.9	5.8	44.4	4.9	48.8	4.9	44.5	5.3
Total # elevated scores M (SD)	1.0 (1.6)		2.4 (2.1)		2.2 (2.3)		4.3 (2.5)		1.6 (1.8)		3.4 (2.5)	

*N* = 359 (Group 1 = 34, Group 2 = 28, Group 3 = 88, Group 4 = 101, Group 5 = 58, Group 6 = 50). ^1^*p* < 0.001 for all measures except anxiety (*p* = 0.011; ANCOVA using PCLC Subtotal as a covariate); ^a^lower scores on these subtests reflect worse outcome. TBI, traumatic brain injury; PTSD, Post-traumatic stress disorder; NIC, Non-injured controls; TBI, Uncomplicated mild TBI; STBI, combines severe TBI, moderate TBI, and complicated mild TBI; TBI-QOL, Traumatic Brain Injury Quality of Life measure; Emot/Behav Dys, Emotional and Behavioral Dyscontrol; Social Participation, Ability to Participate in Social Roles and Activities. HFF, Healthy Family Functioning; UnHFF, Unhealthy Family Functioning.

Select pairwise comparisons (Table 3) to examine the influence of family functioning on HRQOL outcomes within the NIC, MTBI, and STBI groups separately (Hypothesis 1) revealed that overall (i.e., Total Number of Elevated Scores), participants with UnHFF had a significant and meaningfully (i.e., *p* < 0.05 and $\eta_{p}^{2}$ ≥0.06) higher number of clinically elevated HRQOL scores compared to those with HFF in the MTBI (*p* < 0.001, $\eta_{p}^{2}$ =0.07) and STBI (*p* = 0.001, $\eta_{p}^{2}$ =0.10) groups, but not in the NIC group (*p* = 0.107, $\eta_{p}^{2}$ =0.04). Nonetheless, in the NIC group, significant and meaningful differences were found on two of the 10 HRQOL measures (i.e., Depression and Social Participation). In the MTBI group, significant and meaningful differences were also found on two of the 10 HRQOL measures (i.e., Depression and Emotional and Behavioral Dyscontrol). In the STBI group, statistical and meaningful differences were found on seven of the 10 HRQOL measures (all HRQOL measures except Anxiety, Fatigue, and Cognition-General Concerns).

**Table 3:** Select pairwise comparisons across health-related quality of life scores: influence of family functioning within groups.

Health-related quality of life measures	NIC HFF vs. UnHFF (Group 1 v 2)		MTBI HFF vs. UnHFF (Group 3 v 4)		STBI HFF vs. UnHFF (Group 5 v 6)	
	*p* ^1^	$\eta_{p}^{2}$	*p* ^1^	$\eta_{p}^{2}$	*p* ^1^	$\eta_{p}^{2}$
Anxiety	0.501	0.01	0.003	0.05	0.166	0.02
Depression	0.001	0.16	<0.001	0.07	0.002	0.09
Anger	0.397	0.01	0.002	0.05	0.011	0.06
Emot/Behav Dys	0.202	0.03	0.001	0.06	<0.001	0.13
Fatigue	0.383	0.01	0.010	0.04	0.036	0.04
Sleep disturbance	0.449	0.01	0.013	0.03	0.001	0.10
Headaches	0.934	0.00	0.274	0.01	0.009	0.06
Pain interference	0.060	0.06	0.027	0.03	0.001	0.10
Cognition-general concerns	0.365	0.01	0.036	0.02	0.029	0.05
Social participation	0.009	0.11	0.032	0.03	0.001	0.11
Total number of elevated scores	0.107	0.04	<0.001	0.07	0.001	0.10

*N* = 359 (Group 1 = 34, Group 2 = 28, Group 3 = 88, Group 4 = 101, Group 5 = 58, Group 6 = 50). ^1^*p*-value for Family Functioning group after controlling for PTSD symptoms using PCLC Subtotal as a covariate. TBI, traumatic brain injury; NIC, non-injured controls; TBI, uncomplicated mild TBI; STBI, combines severe TBI, moderate TBI, and complicated mild TBI; Emot/Behav Dys, Emotional and Behavioral Dyscontrol; Social Participation, Ability to Participate in Social Roles and Activities; HFF=Healthy Family Functioning; UnHFF=Unhealthy Family Functioning. η_p^2, Partial Eta Squared effect size (0.01 = small, 0.06 = medium, 0.14 = large).

Select pairwise comparisons (Table 4) to examine the influence of healthy family functioning on HRQOL outcomes from TBI (Hypothesis 2) revealed that overall (i.e., Total Number of Elevated Scores), there were no significant and meaningful differences in the total number of clinically elevated HRQOL scores when comparing the MTBI and STBI HFF groups (i.e., Group 3 and Group 5) to both the HFF and UnHFF NIC groups (i.e., Group 1 and Group 2; *p* = 0.074 to 0.841). However, when examining the individual HRQOL measures, a handful of significant and meaningful differences were observed. When compared to the NIC UnHFF group (Group 2), the MTBI HFF group (Group 3) had significant and meaningfully worse scores on Headaches, and the STBI HFF group (Group 5) had worse scores on Social Participation. When compared to the NIC HFF group (Group 1), the MTBI HFF group (Group 3) had significant and meaningfully worse scores on Headaches and Cognition-General Concerns, and the STBI HFF group (Group 5) had worse scores on Cognition-General Concerns.

**Table 4:** Select pairwise comparisons across health-related quality of life scores: influence of healthy family functioning on outcome from traumatic brain injury.

Health-related quality of life measures	MTBI HFF vs. NIC UnHFF (Group 2 v 3)		STBI HFF vs. NIC UnHFF (Group 2 v 5)		MTBI HFF vs. NIC HFF (Group 1 v 3)		STBI HFF vs. NIC HFF (Group 1 v 5)	
	*p* ^1^	$\eta_{p}^{2}$	*p* ^1^	$\eta_{p}^{2}$	*p* ^1^	$\eta_{p}^{2}$	*p* ^1^	$\eta_{p}^{2}$
Anxiety	0.928	0.00	0.678	0.00	0.583	0.00	0.254	0.02
Depression	0.026	0.04	0.077	0.04	0.126	0.02	0.105	0.03
Anger	0.953	0.00	0.995	0.00	0.483	0.00	0.384	0.01
Emot/Behav Dys	0.345	0.01	0.735	0.00	0.684	0.00	0.316	0.01
Fatigue	0.424	0.01	0.942	0.00	0.115	0.02	0.264	0.01
Sleep disturbance	0.717	0.00	0.187	0.02	0.357	0.01	0.525	0.01
Headaches	0.003	0.07	0.242	0.02	0.005	0.07	0.305	0.01
Pain interference	0.732	0.00	0.044	0.05	0.015	0.05	0.892	0.00
Cognition-general concerns	0.049	0.03	0.131	0.03	0.005	0.07	0.007	0.08
Social participation	0.107	0.02	0.027	0.06	0.290	0.01	0.443	0.01
Total number of elevated scores	0.706	0.00	0.841	0.00	0.074	0.03	0.107	0.03

*N* = 359 (Group 1 = 34, Group 2 = 28, Group 3 = 88, Group 4 = 101, Group 5 = 58, Group 6 = 50). ^1^*p*-value for Family Functioning group after controlling for PTSD symptoms using PCLC Subtotal as a covariate. TBI, traumatic brain injury; NIC, non-injured controls; TBI, uncomplicated mild TBI; STBI, combines severe TBI, moderate TBI, and complicated mild TBI; Emot/Behav Dys, Emotional and Behavioral Dyscontrol; Social Participation, Ability to Participate in Social Roles and Activities; HFF, Healthy Family Functioning; UnHFF, Unhealthy Family Functioning. η_p^2 = Partial Eta Squared effect size (0.01 = small, 0.06 = medium, 0.14 = large).

Select pairwise comparisons (Table 5) to examine the influence of unhealthy family functioning on HRQOL outcomes from TBI (Hypothesis 3) revealed that overall (i.e., Total Number of Elevated Scores), the MTBI and STBI UnHFF groups (i.e., Group 4 and Group 6) had a significant and meaningfully higher number of clinically elevated HRQOL scores when compared to the NIC HFF group (Group 1; effect sizes $\eta_{p}^{2}$ =0.14 and 0.18 respectively). When examining the individual HRQOL measures, significant and meaningful differences were found on all 10 HRQOL measures, with effect sizes ranging from medium-large to very large ( $\eta_{p}^{2}$ =0.07 to 0.25). Similarly, the MTBI and STBI UnHFF groups (i.e., Group 4 and Group 6), had a significant and meaningfully higher number of clinically elevated TBI-QOL scores when compared to the NIC UnHFF group (Group 2; effect sizes $\eta_{p}^{2}$ =0.07 for both). When examining the individual HRQOL measures, significant and meaningful differences were found on four of the 10 HRQOL measures when comparing MTBI UnHFF vs. NIC UnHFF groups (Fatigue, Headaches, Pain Interference, Cognition-General Concerns), and on three of the 10 measures when comparing STBI UnHFF vs. NIC UnHFF groups (Emotional and Behavioral Dyscontrol, Headaches, Cognition-General Concerns).

**Table 5:** Select pairwise comparisons across health-related quality of life scores: influence of unhealthy family functioning on outcome from traumatic brain injury.

Health-related quality of life measures	MTBI UnHFF vs. NIC HFF (Group 1 v 4)		STBI UnHFF vs. NIC HFF (Group 1 v 6)		MTBI UnHFF vs. NIC UnHFF (Group 2 v 4)		STBI UnHFF vs. NIC UnHFF (Group 2 v 6)	
	*p* ^1^	$\eta_{p}^{2}$	*p* ^1^	$\eta_{p}^{2}$	*p^1^*	$\eta_{p}^{2}$	*p* ^1^	$\eta_{p}^{2}$
Anxiety	<0.001	0.10	0.011	0.08	0.012	0.05	0.081	0.04
Depression	<0.001	0.15	<0.001	0.21	0.424	0.01	0.325	0.01
Anger	0.001	0.09	0.003	0.11	0.026	0.04	0.071	0.04
Emot/Behav Dys	0.002	0.07	<0.001	0.20	0.102	0.02	0.007	0.09
Fatigue	<0.001	0.12	0.003	0.10	0.004	0.07	0.042	0.05
Sleep disturbance	0.001	0.08	0.016	0.07	0.009	0.05	0.139	0.03
Headaches	<0.001	0.12	0.001	0.13	<0.001	0.12	<0.001	0.15
Pain interference	<0.001	0.16	0.006	0.09	0.008	0.06	0.244	0.02
Cognition-general concerns	<0.001	0.19	<0.001	0.25	<0.001	0.13	<0.001	0.19
Social participation	<0.001	0.10	<0.001	0.15	0.680	0.00	0.433	0.01
Total number of elevated scores	<0.001	0.14	<0.001	0.16	0.003	0.07	0.018	0.07

*N* = 359 (Group 1 = 34, Group 2 = 28, Group 3 = 88, Group 4 = 101, Group 5 = 58, Group 6 = 50). ^1^*p*-value for Family Functioning group after controlling for PTSD symptoms using PCLC total 1–8 as a covariate. TBI, traumatic brain injury; NIC, non-injured controls; TBI, uncomplicated mild TBI; STBI, combines severe TBI, moderate TBI, and complicated mild TBI; Emot/Behav Dys, Emotional and Behavioral Dyscontrol; Social Participation, Ability to Participate in Social Roles and Activities; HFF, Healthy Family Functioning; UnHFF, Unhealthy Family Functioning. $\eta_{p}^{2}$ = Partial Eta Squared effect size (0.01 = small, 0.06 = medium, 0.14 = large).

Using 3-or-more clinically elevated HRQOL scores as a criterion for poor outcome (indicated as a percentage of each group in the following text), the MTBI UnHFF group (69.3%) was (a) 9.8 times more likely to have poor HRQOL outcome when compared to the NIC UnHFF group (35.7%), (b) 28.1 times more likely to have poor HRQOL outcome when compared to the NIC HFF group (17.6%), and (c) 13.2 times more likely to have poor HRQOL outcome when compared to both NIC groups combined (25.8%). Similarly, the STBI UnHFF group (56.0%) was (a) 5.9 times more likely to have poor HRQOL outcome when compared to the NIC UnHFF group, (b) 16.9 times more likely to have poor HRQOL outcome when compared to the NIC HFF group, and (c) 8.1 times more likely to have poor HRQOL outcome when compared to both NIC groups combined.

## Discussion

In support of the first hypothesis, unhealthy family functioning was associated with worse HRQOL in the NIC, MTBI, and STBI groups separately. However, it is important to appreciate that the influence of unhealthy family functioning was strongest in the MTBI and STBI groups. Participants living in an unhealthy family environment following an uncomplicated mild, complicated mild, moderate, severe, or penetrating TBI consistently reported a higher number of elevated HRQOL measures compared to those participants living in a healthy family environment. In contrast, although the influence of an unhealthy family environment on the HRQOL measures was present in the absence of a TBI (i.e., NIC group), the effect of family functioning was far less pronounced and only associated with a handful of HRQOL measures.

In support of the second hypothesis, healthy family functioning was associated with good long-term HRQOL outcomes from TBI. TBI participants living in a healthy family environment reported HRQOL outcomes (i.e., total number of elevated HRQOL measures) that were similar to non-injured controls living in both a healthy or unhealthy family environment. That said, a handful of differences were noted where some of the TBI groups had worse scores on measures of headache and cognitive concerns. However, the effect sizes of these comparisons were generally small in comparison to the effect sizes found for the TBI unhealthy family functioning groups discussed below.

In support of the third hypothesis, unhealthy family functioning was associated with poor long-term HRQOL outcomes from TBI. Overall, TBI participants living in an unhealthy family environment reported worse HRQOL outcomes compared to non-injured controls. The negative influence of living in an unhealthy family environment following MTBI and STBI was generally very strong and was associated with all the HRQOL measures examined. The exception to this was when comparing the MTBI and STBI UnHFF groups to the NIC UnHFF group where the effect was diminished. Nonetheless, both the MTBI and STBI UnHFF groups had a higher number of elevated HRQOL scores compared to the NIC UnHFF group. In addition, using odds ratios, we found that the MTBI UnHFF group was 10 to 28 times more likely to have poor HRQOL outcome depending on the comparison group used (i.e., NIC HFF, NIC UnHFF, or NICs combined). Similarly, the STBI UnHFF group was 6 to 17 times more likely to have poor HRQOL outcome depending on the comparison group (note that a limitation of the odds ratio analyses is that we were unable to control for PTSD symptoms). Taken together, these results are alarming!

It is important to consider the findings within the context of recovery from TBI and comorbidity. SMVs in the MTBI group were on average 15 years post-injury. Recovery from an uncomplicated MTBI is generally anticipated within a few weeks or months. In the military, comorbid conditions can confound chronic self-reported symptoms, regardless of TBI severity. Neurobehavioral and HRQOL symptoms often overlap with symptoms associated with many other non-TBI clinical conditions, commonly PTSD, depression, pain, headaches, sleep disorders, and substance use (11, 12). Research has found that TBI severity has little association with individual, couple, and family level distress in adult and child members in military families (47–49). When an effect was found, worse outcomes were reported in family members of SMVs with a MTBI compared to more severe TBI (47). In contrast, the SMV’s neurobehavioral symptoms have been consistently associated with individual, couple, and family level distress in military families (19, 20, 25, 49, 50). This effect was primarily accounted for by neurobehavioral symptoms related to adjustment (e.g., anxiety, depression, aggression, pain, headaches, fatigue, social, and relationships), but less so neurobehavioral symptoms related to ability (e.g., mobility, vision, speech, memory, attention and concentration) (19, 20, 51). Consistent with previous research, the current study found a stronger association between HRQOL symptoms and family dysfunction in SMVs with a MTBI. Neurobehavioral and HRQOL symptoms are less observable, and thus, more ambiguous in their origin (13). Without obvious physical impairment, it can be challenging for family members to understand why the SMV is experiencing difficulty resuming family roles and reconnecting emotionally following a TBI, particularly when the TBI was sustained during a deployment and confounded by co-occurring psychological conditions (e.g., PTSD). A MTBI is generally associated with less observable physical and neurological impairment, and therefore, greater potential for ambiguity. This ambiguity may lead to greater disruption in family dynamics.

When the TBI groups with unhealthy family functioning were compared to non-injured controls with health family functioning, large effects sizes were found across the four physical, psychological, social, and cognitive HRQOL outcome domains. From a provider perspective, large effects sizes may have clinical meaning for TBI treatment programs. Using Depression as an illustration, large effect sizes were found for Depression between groups, regardless of TBI severity. In a clinical context, a larger effect size means a more substantial clinical impact. In this case, family functioning has a meaningful clinical effect with depression. If a SMV is participating in a clinical intervention for depressive symptoms, and returns to their home environment with unhealthy family functioning, the home environment may undermine treatment outcomes if improving family functioning is not part of treatment goals. It is important to also consider that for the remaining comparisons medium effects were observed. While the magnitude of the effect was smaller, a medium effect may still have notable clinical relevance and should not be overlooked in a clinical setting. A small effect size is generally the least clinically meaningful. However, a small effect size must be considered in relation the condition and potential risk. For example, a small effect size may be indicative of the start of a subclinical condition that is trending in a negative clinical trajectory in future. In some cases, a small effect can have large consequences, such as expressions of aggressive or homicidal behavior in family and social settings. In a clinical setting, it is also important to address the array of clinical needs, not just a single need, otherwise, the remaining unmet needs may outweigh any benefits gained. In previous research, having health care needs and unmet needs were associated with worse health outcomes in military families with warfighter TBI (52, 53). The cumulative effect of needs was associated with worse health outcomes compared to a single need.

It is important to note that the directionality of the relationship between family functioning and recovery from TBI in the current study was not explored. Research has demonstrated that unhealthy family relationships are likely to be an interaction of bidirectional and reciprocal individual, couple, and family factors; i.e., individual, couple, and family distress are likely to influence and be influenced by each other. Unhealthy family functioning and chronic symptoms post-TBI have been related to poor individual health outcomes in the SMV’s family members (19, 20, 25, 48, 54), regardless of TBI severity (47). Family members often feel anxious and vigilant trying to navigate and control the SMV’s mood and behavior (50, 55). SMVs experiencing withdrawal and avoidance can appear emotionally disengaged toward family members, impacting intimate relationships in couples and parent–child bonds in their children (20, 48). Family members often adopt behavioral and emotional actions, or accommodations, in an attempt to alleviate the SMV’s distress and reduce conflict, such as avoiding physical and emotional intimacy, contentious conversations, social situations, and household noise, and assuming household chores, roles, and responsibilities previously shared with the SMV (56, 57). While often, but not always well-intentioned, behavioral and emotional accommodations may inadvertently reinforce or facilitate the SMV’s symptoms, undermine treatment goals, and impede recovery and fitness for duty outcomes. Accommodations have also been related to increased psychological, social, caregiving, relationship, and family distress in family members (56–58). Structural equation modeling or two stage least squares would be used to explore reciprocal relationships. These types of multivariate structural relationships require larger sample sizes than the current study. Reciprocal models additionally require theoretical driven instrumental variables to work mathematically. Instrumental variables need to be defined in advance, not identified statistically. More research exploring dyadic relationships in military couples will lead to the formulation of a theory based on these observed patterns that can be further tested and refined using reciprocal modeling statistical methods in future.

SMVs with chronic symptoms following a TBI often require informal care and support from family members, most frequently an intimate partner or parent, but also their children (59–62). Caregiving strain (or burden) has been consistently associated with distress in adult family members caring for SMVs with TBI (25, 49, 63–65). Intimate partner caregivers often report changes in the dynamic of their relationship with the SMV, navigating both care provision and romantic roles (55). Higher levels of caregiving distress have been associated with family dysfunction, relationship dissatisfaction, and divorce considerations in intimate partners (19, 20, 66, 67). No longer being in an intimate relationship with the SMV was one of the most frequent reasons reported when no longer caregiving for the SMV (68). Economic strain has also been reported in TBI military caregiving families, such as employment absenteeism, leaving the labor force, out-of-pocket expenses, depleting assets, and accumulated debt (49, 59, 69, 70). Reduced employment and earnings in the present can affect the amount of social security, Medicare benefits, 401(k) and other retirement plan contributions received at retirement age, potentially impacting financial stability in military caregiving families over time (71).

Family caregivers often report that care provision creates tension in the household and impacts their parenting and parent–child relationships (72). Worse physical, psychological, social, and academic outcomes in children of SMVs with TBI in military caregiving homes has been reported (48, 54, 73). Worse outcomes have been related to higher levels of parental distress (48, 74). Children often report that their own health issues go unnoticed due to the demands of caregiving (75). Children may also take on caregiving responsibilities that other children their age likely do not, leaving limited personal time for education, recreation, and social activities (72, 75). High levels of distress have been found in young adult children in TBI military caregiving families (48). Young adulthood is a period where an interruption in health care may be experienced during the transition from parental guardianship to independence, and from child to adult health care systems (76). This is particularly important for children of SMVs who served during the post-9/11 conflicts; a military period notorious for frequent relocations, lengthy combat deployments, blast-related polytrauma injuries, and long-term caregiving support from family members (48, 77). The number of children from military families who served in the post-9/11 conflicts approaching the age of transition into adulthood is increasing.

The estimated yearly value of care provision by military family members in 2014 was 14 billion USD, resulting in direct benefits to society in defrayed health care costs (72). If family members are no longer able or willing to provide support, advocacy, and care, the SMV would need to find alternative assistance from military and public health care systems and community programs. These costs, along with the costs to attend to their own care needs, would also ultimately confer on society. If alternative support is not secured, it places the SMV at risk for adverse outcomes, such as deteriorating health, homelessness, suicide, violence, and crime. A holistic, family-centered interdisciplinary model of care for TBI treatment is recommended for military families.

The influence of family on warfighter recovery and readiness is a widely acknowledged concept, though woefully under researched and understood. In 2021, the United States Department of Defense released the Warfighter Brain Health Initiative Strategy and Action Plan. In that plan, an emphasis was placed on the need to combat the effects of brain injury on warfighters and their families, and better address their brain health needs. The need to further understand long-term and late effects that prevent warfighters from returning to optimal brain health following TBI was identified as a line of effort. An important and unique addition to this initiative was the finding that high levels of distress and dysfunction in a warfighter’s home environment are likely to be a strong risk factor for poor recovery, return to duty, and long-term brain health outcomes following a TBI. It is possible that when warfighters recover in an unhealthy environment, their recovery and return to duty may be compromised because their family members and home environment are unable to provide the care and support needed. Poor recovery may increase the probability that the service member may require a medical separation or may return to the operational environment with reduced capabilities. A warfighter’s recovery and return to duty may be enhanced if their family members and home environment are healthy and cohesive, and members work together to facilitate recovery and return to duty. In recent research, intimate partners of warfighters who were receiving treatment at the National Intrepid Center of Excellence (NICoE) Intensive Outpatient Program (IOP) for chronic symptoms following MTBI reported a worsening longitudinal trend in clinically elevated scores on physical, psychological, social, and caregiving HRQOL measures (78). Elevated symptoms on these same measures were identified as strong intimate partner risk factors for chronic neurobehavioral and HRQOL symptoms in SMVs with TBI (21, 79, 80). Family dynamics may have important implications for military TBI treatment programs. If a warfighter is discharged from treatment to a home environment with high levels of distress and dysfunction, improvement in symptoms and return to duty outcomes may diminish over time, because conflict, disorganization, and poor affective, behavioral, and social regulation within their family may undermine treatment outcomes.

SMVs with TBI, PTSD, and other polytrauma have stated a preference to have their intimate partner and children involved in their therapy over individual treatment due to its impact on the quality of the SMV’s family relationships and quality of life (81, 82). Success has been shown in reducing individual, couple, and family-level distress in military families who participated in the Families OverComing Under Stress (FOCUS) resiliency training program (24, 83). FOCUS is a training program that teaches practical skills to help military families respond to and cope with stress and change related to military life and trauma. FOCUS is established at United States military installations and also provides an interactive online platform. FOCUS may be helpful for military families navigating chronic symptoms and treatment goals in warfighters with TBI.

Meta-analysis has highlighted improvement in SMVs with TBI after participation in cognitive rehabilitation (84). Some researchers have started including intimate partners in cognitive rehabilitations programs using a couple-based or conjoint intervention design to address health and family issues in military couples as a dyadic approach to treatment outcomes. *Cognitive-Behavioral Conjoint Therapy* (CBCT) is a couples therapy using dyadic cognitive-behavioral approaches for PTSD. CBCT has been helpful in reducing psychological and relationship distress, social and communication avoidance, and use of accommodations in military couples with warfighter PTSD (85–87). An online, guided, self-help adaptation of CBCT, *Couple Helping Overcome PTSD and Enhance Relationships* (HOPES), has also been found to be effective in reducing PTSD symptoms, relationship distress, and use of accommodations (88). Dyadic cognitive-behavioral approaches may be helpful for improving HRQOL, return to duty, and readiness outcomes in military couples with SMV TBI.

In response to the growing body of research demonstrating the bidirectional associations between warfighter symptoms post-TBI and individual, couple, and family distress in military families, members of our Caregiver and Family Member Study^1^ team recently established a Family Wellness Program (FWP) (89) for intimate partners of warfighters receiving treatment in the NICoE IOP. IPs complete a set of measures to screen for elevated physical, psychological, social, and caregiving distress, and then consult with a clinician to discuss their responses and assess potential treatment or referral needs during the family week (fourth week) of the IOP. IPs are provided with a brief clinical report, including an interpretation of their symptom severity on each measure and recommendations for clinical follow up. The FWP is also expanding operations across the Defense Intrepid Network (DIN) Intrepid Spirit Centers (ISC) and integrating couple-based, conjoint cognitive-behavioral therapy referenced above.

Several potential limitations are worth mentioning. First, the small sample sizes of some groups (e.g., Group 1, *n* = 34; Group 2, *n* = 28) may have undermined statistical power and true differences between some group comparisons may have been missed. Second, the current study examined family functioning and HRQOL outcomes at a single point in time rather than multiple time points over the recovery trajectory. Longitudinal research is recommended to allow for multiple evaluations and a better assessment of change in family functioning and TBI outcomes over time. Third, certain factors that may have accounted for family functioning and TBI outcomes were not included in the current study, such as pre-existing factors, coping styles, community and military supports, participation in rehabilitation and other treatment programs, and barriers to health care. Finally, the use of United States military members may limit the generalizability of the findings to military members in countries other than the United States and also non-military populations.

In sum, this study demonstrated that unhealthy family functioning was strongly associated with poor long-term HRQOL outcomes following TBI of all severities. The association between unhealthy family functioning and chronic symptoms post-TBI may have significant implications for acute and chronic warfighter brain health. A service member will likely be given the best chance for recovery within a well-functioning family. The negative influence of family distress on warfighter brain health is a factor that is under-appreciated, modifiable, and has the potential for significant impact on warfighter recovery and readiness. One family member noted that *“Far too often, the veteran is the only one who is made to be the primary person in need of support. If you are not talking about the other people in their life, you are missing a giant piece of the puzzle”* (p. 16) (75). A holistic approach to TBI treatment that focuses on how individual, couple, and family factors interact with each other will likely maximize warfighter brain health, Department of Defense retention of mission-critical support personnel, and outcomes for military families overall. The establishment of the FWP at the NICoE and expansion across the DIN will open the door for family wellness to have a long-term place in DoD TBI treatment programs as a holistic, family-centered interdisciplinary model of care for warfighter brain health and return to duty following a TBI, and healthy, resilient, and military ready families.

## Data Availability

The datasets presented in this article are not readily available because the data will be made available through the U. S. Federal Interagency Traumatic Brain Injury Research (FITBIR) informatics system in future. Requests to access the datasets should be directed to https://fitbir.nih.gov/.

## Footnotes

^1^The Defense and Veterans Brain Injury Center-Traumatic Brain Injury Center of Excellence (DVBIC-TBICoE) 15-Year Longitudinal TBI Study consists of two independent studies: (1) the Natural History of TBI Study and (2) the Caregiver and Family Member Study. Both address elements in Section 721 of the John Warner National Defense Authorization Act for Fiscal Year 2007 (Public Law 1,009–364). The current sample included participants from the Natural History of TBI Study.

## References

[1] Cozza SJ Guimond JM. Working with combat-injured families through the recovery trajectory In: Wadsworth SM Riggs D, editors. Risk and resilience in US military families. New York: Springer Science and Business Media (2011). 259–80.

[2] Traumatic Brain Injury Center of Excellence. *DoD numbers for traumatic brain injury*. (2024). Available online at: www.health.mil (Accessed September 27, 2024).

[3] Lange RT Brickell TA French LM Merritt VC Bhagwat A Pancholi S. Neuropsychological outcome from uncomplicated mild, complicated mild, and moderate traumatic brain injury in US military personnel. Arch Clin Neuropsychol. (2012) 27:480–94. doi: 10.1093/arclin/acs059, PMID: 22766317

[4] de Guise E Lepage J Tinawi S LeBlanc J Dagher J Lamoureux J. Comprehensive clinical picture of patients with complicated vs uncomplicated mild traumatic brain injury. Clin Neuropsychol. (2010) 24:1113–30. doi: 10.1080/13854046.2010.506199, PMID: 20730678

[5] Williams DH Levin HS Eisenberg HM. Mild head injury classification. Neurosurgery. (1990) 27:422–8. doi: 10.1227/00006123-199009000-00014, PMID: 2234336

[6] Voormolen DC Zeldovich M Haagsma JA Polinder S Friedrich S Maas AIR. Outcomes after complicated and uncomplicated mild traumatic brain injury at three- and six-months post-injury: results from the CENTER-TBI study. J Clin Med. (2020) 9:1525. doi: 10.3390/jcm9051525, PMID: 32443573 PMC7291134

[7] Lange RT Brickell TA Ivins B Vanderploeg RD French LM. Variable, not always persistent, postconcussion symptoms after mild TBI in US military service members: a five-year cross-sectional outcome study. J Neurotrauma. (2013) 30:958–69. doi: 10.1089/neu.2012.2743, PMID: 23205671

[8] Lange RT Lippa SM Bailie JM Wright M Driscoll A Sullivan J. Longitudinal trajectories and risk factors for persistent postconcussion symptom reporting following uncomplicated mild traumatic brain injury in U.S. military service members. Clin Neuropsychol. (2020) 34:1134–55. doi: 10.1080/13854046.2020.1746832, PMID: 32284000

[9] Hai T Agimi Y Stout K. Prevalence of comorbidities in active and reserve service members pre and post traumatic brain injury, 2017-2019. Mil Med. (2023) 188:e270–7. doi: 10.1093/milmed/usab342, PMID: 34423819 PMC9825245

[10] Brickell TA Wright MM Sullivan JK Varbedian NV Nose KA Rather LM. Caregiver sleep impairment and service member and veteran adjustment following traumatic brain injury is related to caregiver health-related quality of life. J Clin Sleep Med. (2022) 18:2577–88. doi: 10.5664/jcsm.10164, PMID: 35912703 PMC9622982

[11] Lange RT French LM Lippa SM Ballie JM Brickell TA. Post-traumatic stress disorder is a stronger predictor of long-term neurobehavioral outcome than traumatic brain injury severity. J Trauma Stress. (2020) 33:318–29. doi: 10.1002/jts.22480, PMID: 32379932

[12] Lange RT Lippa SM French LM Bailie JM Gartner RL Driscoll AE. Long-term neurobehavioural symptom reporting following mild, moderate, severe, and penetrating traumatic brain injury in U.S. military service members. Neuropsychol Rehabil. (2020) 30:1762–85. doi: 10.1080/09602011.2019.1604385, PMID: 31003592

[13] Gorman LA Fitzgerald HE Blow AJ. Parental combat injury and early child development: a conceptual model for differentiating effects of visible and invisible injuries. Psychiatry Q. (2010) 81:1–21. doi: 10.1007/s11126-009-9116-4, PMID: 19941074

[14] Sayers SL Farrow VA Ross J Oslin DW. Family problems among recently returned military veterans referred for a mental health evaluation. J Clin Psychiatry. (2009) 70:163–70. doi: 10.4088/jcp.07m03863, PMID: 19210950

[15] Bui E Zakarian RJ Laifer LM Sager JC Chen Y Cohen S. Psychometric properties of the parenting sense of competence scale in treatment-seeking post-9/11 veterans. J Child Fam Stud. (2017) 26:464–70. doi: 10.1007/s10826-016-0580-9

[16] Laifer LM Blackburn AM Goetter EM Ohye BY Simon NM Bui E. Potential mediating role of parenting competence in the relationship between posttraumatic stress disorder and family functioning in post-9/11 veteran parents. J Child Fam Stud. (2019) 28:1843–9. doi: 10.1007/s10826-019-01405-9

[17] Makhija NJ Ohye BY Zakarian RJ Jakubovic RJ Bui E. Contributions of parenting sense of competence to family functioning in a sample of military-connected families living in the community. Fam J. (2019) 27:404–8. doi: 10.1177/1066480719868700

[18] Pugh MJ Swan AA Carlson KF Jaramillo CA Eapen BC Dillahunt-Aspillaga C. Traumatic brain injury severity, comorbidity, social support, family functioning, and community reintegration among veterans of the Afghanistan and Iraq wars. Arch Phys Med Rehabil. (2018) 99:S40–9. doi: 10.1016/j.apmr.2017.05.021, PMID: 28648681

[19] Brickell TA French LM Sullivan JK Varbedian NV Wright MM Lange RT. Unhealthy family functioning is associated with poor health-related quality of life in spouse caregivers of service members and veterans following traumatic brain injury. Psychol Trauma Theory Res Pract Policy. (2022) 14:587–96. doi: 10.1037/tra0001055 34323566

[20] Brickell TA French LM Varbedian NV Sewell JM Schiefelbein FC Wright MM. Relationship satisfaction among spouse caregivers of service members and veterans with comorbid mild traumatic brain injury and post-traumatic stress disorder. Fam Process. (2022) 61:1525–40. doi: 10.1111/famp.12731, PMID: 34859431

[21] Brickell TA Ivins BJ Wright MM Sullivan JK Baschenis SM French LM. Intimate partner distress is strongly associated with worse warfighter brain health following mild traumatic brain injury. Psychol Trauma Theory Res Pract Policy. (2025) 1:1889. doi: 10.1037/tra0001889, PMID: 40193486

[22] Corrigan JD Bogner J. Initial reliability and validity of the Ohio State University TBI identification method. J Head Trauma Rehabil. (2007) 22:318–29. doi: 10.1097/01.HTR.0000300227.67748.77, PMID: 18025964

[23] Epstein NB Baldwin LM Bishop DS. The McMaster family assessment device. J Marital Fam Ther. (1983) 9:171–80. doi: 10.1111/j.1752-0606.1983.tb01497.x

[24] Lester P Liang LJ Milburn N Mogil C Woodward K Nash W. Evaluation of a family-centered preventive intervention for military families: parent and child longitudinal outcomes. J Am Acad Child Adolesc Psychiatry. (2016) 55:14–24. doi: 10.1016/j.jaac.2015.10.009, PMID: 26703905

[25] Griffin JM Lee MK Bangerter LR Van Houtven CH Friedemann-Sánchez G Phelan SM. Burden and mental health among caregivers of veterans with traumatic brain injury/polytrauma. Am J Orthopsychiatry. (2017) 87:139–48. doi: 10.1037/ort0000207, PMID: 28206801

[26] Wilde EA Whiteneck GG Bogner J Bushnik T Cifu DX Dikmen SS. Recommendations for the use of common outcome measures in traumatic brain injury research. Arch Phys Med Rehabil. (2010) 91:1650–60.e17. doi: 10.1016/j.apmr.2010.06.033 21044708

[27] Ponsford J Olver J Ponsford M Nelms R. Long-term adjustment of families following traumatic brain injury where comprehensive rehabilitation has been provided. Brain Inj. (2003) 17:453–68. doi: 10.1080/0269905031000070143, PMID: 12745702

[28] Mansfield AK Keitner GI Dealy J. The family assessment device: an update. Fam Process. (2015) 54:82–93. doi: 10.1111/famp.12080, PMID: 24920469

[29] Miller IW Epstein NB Bishop DS Keitner GI. The McMaster family assessment device: reliability and validity. J Marital Fam Ther. (1985) 11:345–56. doi: 10.1111/j.1752-0606.1985.tb00028.x

[30] Tulsky DS Kisala PA Victorson D Carlozzi N Bushnik T Sherer M. TBI-QOL: development and calibration of item banks to measure patient reported outcomes following traumatic brain injury. J Head Trauma Rehabil. (2016) 31:40–51. doi: 10.1097/HTR.0000000000000131, PMID: 25931184 PMC4697960

[31] Gershon RC Lai JS Bode R Choi S Moy C Bleck T. Neuro-QOL: quality of life item banks for adults with neurological disorders: item development and calibrations based upon clinical and general population testing. Qual Life Res. (2012) 21:475–86. doi: 10.1007/s11136-011-9958-8, PMID: 21874314 PMC3889669

[32] Ataman R Alhasani R Auneau-Enjalbert L Quigley A Michael HU Ahmed S. Measurement properties of the traumatic brain injury quality of life (TBI-QoL) and spinal cord injury quality of life (SCI-QoL) measurement systems: a systematic review. Syst Rev. (2025) 14:18. doi: 10.1186/s13643-024-02722-x, PMID: 39838501 PMC11749626

[33] Merritt VC Brickell TA Bailie JM Hungerford L Lippa SM French LM. Low resilience following traumatic brain injury is strongly associated with poor neurobehavioral functioning in U.S. military service members and veterans. Brain Inj. (2022) 36:339–52. doi: 10.1080/02699052.2022.2034183, PMID: 35171749

[34] Pattinson CL Brickell TA Bailie JM Hungerford LD Lippa SM French LM. Sleep disturbances following traumatic brain injury are associated with poor neurobehavioral outcomes in U.S. military service members and veterans. J Clin Sleep Med. (2021) 17:2425–38. doi: 10.5664/jcsm.9454, PMID: 34216198 PMC8726371

[35] Lange RT Brickell TA Bailie JM Tulsky DS French LM. Clinical utility and psychometric properties of the traumatic brain injury quality of life scale (TBI-QOL) in US military service members. J Head Trauma Rehabil. (2016) 31:62–78. doi: 10.1097/HTR.0000000000000149, PMID: 26716697

[36] VanDer Ploeg RD Cooper DB Belanger HG Donnell AJ Kennedy JE Hopewell CA. Screening for postdeployment conditions: development and cross-validation of an embedded validity scale in the neurobehavioral symptom inventory. J Head Trauma Rehabil. (2014) 29:1–10. doi: 10.1097/HTR.0b013e318281966e, PMID: 23474880

[37] Cicerone KD Kalmar K. Persistent postconcussion syndrome: the structure of subjective complaints after mild traumatic brain injury. J Head Trauma Rehabil. (1995) 10:1–17. doi: 10.1097/00001199-199510030-00002

[38] Armistead-Jehle P Cooper DB Grills CE Cole WR Lippa SM Stegman RL. Clinical utility of the mBIAS and NSI validity-10 to detect symptom over-reporting following mild TBI: a multicenter investigation with military service members. J Clin Exp Neuropsychol. (2018) 40:213–23. doi: 10.1080/13803395.2017.1329406, PMID: 28539077

[39] Lippa SM Lange RT Bailie JM Kennedy JE Brickell TA French LM. Utility of the Validity-10 scale across the recovery trajectory following traumatic brain injury. J Rehabil Res Dev. (2016) 53:379–90. doi: 10.1682/JRRD.2015.01.0009, PMID: 27273336

[40] Blanchard EB Jones-Alexander J Buckley TC Forneris CA. Psychometric properties of the PTSD checklist (PCL). Behav Res Ther. (1996) 34:669–73. doi: 10.1016/0005-7967(96)00033-2, PMID: 8870294

[41] American Psychiatric Association. Diagnostic and statistical manual of mental disorders. 4th ed. Washington, DC: American Psychiatric Association (1994).

[42] Yufik T Simms LJ. A meta-analytic investigation of the structure of posttraumatic stress disorder symptoms. J Abnorm Psychol. (2010) 119:764–76. doi: 10.1037/a0020981, PMID: 21090877 PMC4229035

[43] Cohen J. Stastical power analysis for the behavioral sciences. 2nd ed. Hillsdale, NJ: Lawrence Erlbaum Associates (1988).

[44] Sullivan GM Feinn R. Using effect size - or why the p value is not enough. J Grad Med Educ. (2012) 4:279–82. doi: 10.4300/JGME-D-12-00156.1, PMID: 23997866 PMC3444174

[45] Lakens D. Calculating and reporting effect sizes to facilitate cumulative science: a practical primer for t-tests and ANOVAs. Front Psychol. (2013) 4:863. doi: 10.3389/fpsyg.2013.00863, PMID: 24324449 PMC3840331

[46] Page P. Beyond statistical significance: clinical interpretation of rehabilitation research literature. Int J Sports Phys Ther. (2014) 9:726–36. PMID: 25328834 PMC4197528

[47] Brickell TA Lippa SM Wright MM Varbedian NV Tippett CE Byrd AM. Is traumatic brain injury severity in service members and veterans related to health-related quality of life in their caregivers? J Head Trauma Rehabil. (2022) 37:338–49. doi: 10.1097/HTR.0000000000000802, PMID: 35862894

[48] Brickell TA Wright MM Sullivan JK Varbedian NV Gillow KC Baschenis SM. Health outcomes in children living in military families caring for a service member or veteran with traumatic brain injury. J Child Fam Stud. (2024) 33:908–23. doi: 10.1007/s10826-023-02683-0

[49] Moriarty H Winter L Short TH True G. Exploration of factors related to depressive symptomatology in family members of military veterans with traumatic brain injury. J Fam Nurs. (2018) 24:184–216. doi: 10.1177/1074840718773470, PMID: 29848196

[50] Brickell TA French LM Wright MM Lange RT. Aggression in military members with mild traumatic brain injury and post-traumatic stress disorder is associated with intimate partner health-related quality of life. Womens Health Issues. (2022) 32:526–33. doi: 10.1016/j.whi.2022.04.003, PMID: 35643836

[51] Brickell TA Ivins BJ Wright MM French LM Lange RT. Longitudinal health outcomes in caregivers of military members with traumatic brain injury. Rehabil Psychol. (2024) 69:135–44. doi: 10.1037/rep0000522, PMID: 38127539

[52] Brickell TA Lippa SM French LM Gartner RL Driscoll AE Wright MM. Service needs and health outcomes among caregivers of service members and veterans following TBI. Rehabil Psychol. (2019) 64:72–86. doi: 10.1037/rep0000249, PMID: 30247054

[53] Lange RT French LM Lippa SM Rogers AA Gillow K Tippett CE. Service needs and neurobehavioral functioning following traumatic brain injury in U.S. military personnel. Rehabil Psychol. (2024) 70:63–74. doi: 10.1037/rep0000556, PMID: 38780581

[54] Brickell TA French LM Lippa SM Lange RT. The impact of deployment and traumatic brain injury on the health and behavior of children of US military service members and veterans. Clin Child Psychol Psychiatry. (2018) 23:425–41. doi: 10.1177/1359104517740405, PMID: 29139308

[55] Carlozzi NE Brickell TA French LM Sander A Kratz AL Tulsky DS. Caring for our wounded warriors: a qualitative examination of health-related quality of life in caregivers of individuals with military-related traumatic brain injury. J Rehabil Res Dev. (2016) 53:669–80. doi: 10.1682/JRRD.2015.07.0136, PMID: 27997672 PMC5180206

[56] Renshaw KD Allen ES Fredman SJ Giff ST Kern C. Partners’ motivations for accommodating posttraumatic stress disorder symptoms in service members: the reasons for accommodation of PTSD scale. J Anxiety Disord. (2020) 71:102199. doi: 10.1016/j.janxdis.2020.102199, PMID: 32097730 PMC10733866

[57] Campbell SB Renshaw KD. Daily posttraumatic stress disorder symptom accommodation and relationship functioning in military couples. Fam Process. (2019) 58:908–19. doi: 10.1111/famp.12393, PMID: 30216445 PMC6417979

[58] Campbell SB Renshaw KD. Posttraumatic stress disorder and relationship functioning: a comprehensive review and organizational framework. Clin Psychol Rev. (2018) 65:152–62. doi: 10.1016/j.cpr.2018.08.003, PMID: 30205286 PMC6173976

[59] Brickell TA French LM Lippa SM Lange RT. Characteristics and health outcomes of post-9/11 caregivers of US service members and veterans following traumatic brain injury. J Head Trauma Rehabil. (2018) 33:133–45. doi: 10.1097/HTR.0000000000000384, PMID: 29517593

[60] Griffin JM Friedemann-Sánchez G Jensen AC Taylor BC Gravely A Clothier B. The invisible side of war: families caring for US service members with traumatic brain injuries and polytrauma. J Head Trauma Rehabil. (2012) 27:3–13. doi: 10.1097/HTR.0b013e3182274260, PMID: 21873883

[61] Sander AM Boileau NR Hanks RA Tulsky DS Carlozzi NE. Emotional suppression and hypervigilance in military caregivers: relationship to negative and positive affect. J Head Trauma Rehabil. (2020) 35:E10–20. doi: 10.1097/HTR.0000000000000507, PMID: 31365438 PMC7643713

[62] Moriarty H Winter L True G Robinson K Short TH. Depressive symptomatology mediates associations with community reintegration in veterans with TBI. Mil Psychol. (2016) 28:376–89. doi: 10.1037/mil0000122

[63] Brickell TA French LM Lippa SM Lange RT. Caring for a service member or veteran following traumatic brain injury influences caregiver mental health. Mil Psychol. (2020) 32:341–51. doi: 10.1080/08995605.2020.1754149, PMID: 38536254 PMC10013226

[64] Brickell TA Wright MM Lippa SM Sullivan JK Ballie JM French LM. Resilience is associated with health-related quality of life in caregivers of service members and veterans following traumatic brain injury. Qual Life Res. (2020) 29:2781–92. doi: 10.1007/s11136-020-02529-y, PMID: 32500241

[65] Brickell TA French LM Lippa SM Lange RT. Burden among caregivers of service members and veterans following traumatic brain injury. Brain Inj. (2018) 32:1541–8. doi: 10.1080/02699052.2018.1503328, PMID: 30148407

[66] Skomorovsky A Martynova E Lee JEC Dursun S. Spousal perceptions of military members’ health and their well-being and divorce considerations: the role of caregiver burden. Mil Behav Health. (2017) 5:406–16. doi: 10.1080/21635781.2017.1335256

[67] Griffin JM Friedemann-Sánchez G Carlson KF Jensen AC Gravely A Taylor BC. Resources and coping strategies among caregivers of operation Iraqi freedom (OIF) and operation enduring freedom (OEF) veterans with polytrauma and traumatic brain injury In: Wadsworth SM Riggs DS, editors. Military deployment and its consequences for families. New York: Springer (2014). 259–80.

[68] Brickell TA Wright MM Sullivan JK Varbedian NV Gillow KC Baschenis SM. Longitudinal health-related quality of life in military caregivers no longer providing care. Rehabil Psychol. (2023) 68:396–406. doi: 10.1037/rep0000489, PMID: 37917461

[69] Van Houtven CH Friedemann-Sánchez G Clothier B Levison D Taylor BC Jensen AC. Is policy well-targeted to remedy financial strain among caregivers of severely injured U.S. service members? Inquiry. (2012) 49:339–51. doi: 10.5034/inquiryjrnl_49.04.01, PMID: 23469677

[70] Brickell TA French LM Gartner RL Driscoll AE Wright MM Lippa SM. Factors related to perceived burden among caregivers of service members/veterans following TBI. Rehabil Psychol. (2019) 64:307–19. doi: 10.1037/rep0000272, PMID: 30896245

[71] Miller KEM Lindquist JH Olsen MK Smith VA Voils CI Oddone EZ. Invisible partners in care: snapshot of well-being among caregivers receiving comprehensive support from veterans affairs. Health Sci Rep. (2019) 2:e112. doi: 10.1002/hsr2.112, PMID: 30937391 PMC6427058

[72] Ramchand R Tanielian T Fisher MP Vaughan CA Trail TE Epley C. Hidden heroes: America’s military caregivers. Santa Monica, CA: Rand Corporation (2014). 10.7249/rhq-04-02-14 PMC5052006 28083343

[73] Hisle-Gorman E Susi A Gorman GH. The impact of military parents' injuries on the health and well-being of their children. Health Aff. (2019) 38:1358–65. doi: 10.1377/hlthaff.2019.00276, PMID: 31381386

[74] Brickell TA Wright MM Sullivan JK Ivins BJ Varbedian NV Byrd AM. Longitudinal pediatric symptom trajectories and parental risk factors for psychological distress in children of warfighters with traumatic brain injury. Front Psychiatry. (2025) 2025:801. doi: 10.3389/fpsyt.2025.1465801

[75] Malick S Sandoval M Santiago T Jacobs Johnson C Gehrke A Metallic E. Hidden helpers at the frontlines of caregiving: Supporting the healthy development of children from military and veteran caregiving homes. Princeton, NJ: Mathematica (2021).

[76] Lorenzil M Broemeling AM Glickman V Goddard K Pritchard S Rogers P. *Childhood, adolescent and Young adult Cancer survivors (CAYACS) research program: Results to date*. National Cancer Institute of Canada’s Making Connections: A Canadian Cancer Research Conference; Toronto, Ontario, Canada (2007).

[77] Chandra A Martin LT Hawkins SA Richardson A. The impact of parental deployment on child social and emotional functioning: perspectives of school staff. J Adolesc Health. (2010) 46:218–23. doi: 10.1016/j.jadohealth.2009.10.009, PMID: 20159497

[78] Brickell TA French LM Wright MM Sullivan JK Ivins BA Varbedian NV. Family caregivers of service members in U.S. department of defense health care report impairment in longitudinal health outcomes. Psychol Trauma Theory Res Pract Policy. (2025) 17:406–15. doi: 10.1037/tra0001712, PMID: 38913717

[79] Brickell TA Wright MM Lippa SM Baschenis SM Sullivan JK Hungerford LD. Family risk factors are related to warfighter brain health: a dyad study. Rehabil Psychol. (2025) 1:608. doi: 10.1037/rep0000608, PMID: 40111785

[80] Brickell TA Ivins BJ Wright MM Sullivan JK Baschenis SM Gillow KC. A dyad approach to understanding intimate partner distress as a risk factor for poor warfighter brain health following mild traumatic brain injury in military couples. J Head Trauma Rehabil. (2025) 2025:1060. doi: 10.1097/HTR.0000000000001060 40396896

[81] Ohye BY Brendel RW Fredman SJ Bui E Rauch PK Allard MD. Three-generation model: a family systems framework for the assessment and treatment of veterans with posttraumatic stress disorder and related conditions. Prof Psychol Res Pract. (2015) 46:97–106. doi: 10.1037/a0037735

[82] Meis LA Schaaf KW Erbes CR Polusny MA Miron LR Schmitz TM. Interest in partner-involved services among veterans seeking mental health care from a VA PTSD clinic. Psychol Trauma Theory Res Pract Policy. (2013) 4:334–42. doi: 10.1037/a0028366

[83] Saltzman WR Lester P Milburn N Woodward K Stein J. Pathways of risk and resilience: impact of a family resilience program on active-duty military parents. Fam Process. (2016) 55:633–46. doi: 10.1111/famp.12238, PMID: 27597440

[84] Austin TA Hodges CB Thomas ML Szabo YZ Parr S Eschler BD. Meta-analysis of cognitive rehabilitation interventions in veterans and service members with traumatic brain injuries. J Head Trauma Rehabil. (2024) 39:258–72. doi: 10.1097/HTR.0000000000000924, PMID: 38270528 PMC11227399

[85] Fredman SJ Macdonald A Monson CM Dondanville KA Blount TH Hall-Clark BN. Intensive multi-couple group therapy for PTSD: a non-randomized pilot study with military and veteran dyads. Behav Ther. (2020) 51:700–14. doi: 10.1016/j.beth.2019.10.003, PMID: 32800299 PMC10760800

[86] Fredman SJ Le Y Macdonald A Monson CM Rhoades GK Dondanville KA. A closer examination of relational outcomes from a pilot study of abbreviated, intensive, multi-couple group cognitive-behavioral conjoint therapy for PTSD with military dyads. Fam Process. (2021) 60:712–26. doi: 10.1111/famp.12654, PMID: 33876831 PMC10760895

[87] Pukay-Martin ND Fredman SJ Martin CE Le Y Haney H Sullivan C. Effectiveness of cognitive behavioral conjoint therapy for posttraumatic stress disorder (PTSD) in a U.S. veterans affairs PTSD clinic. J Trauma Stress. (2022) 35:644–58. doi: 10.1002/jts.22781, PMID: 34942022 PMC9035020

[88] Monson CM Wagner AC Crenshaw AO Whitfield KM Newnham CM Valela R. An uncontrolled trial of couple HOPES: a guided online couple intervention for PTSD and relationship enhancement. J Fam Psychol. (2022) 36:1036–42. doi: 10.1037/fam0000976, PMID: 35266773

[89] Brickell TA Wright MM Baschenis SM Lange RT Sullivan JK French LM. The Family Wellness Program: A bench to bedside translation of behavioral and social science research into a clinical program for intimate partners of warfighters following traumatic brain Injury. Frontiers in Health Services. (2025) in press., PMID: 35266773

